# Supplementary material for: Keystone Species and Modularity in Microbial Hydrocarbon Degradation Uncovered by Network Analysis and Association Rule Mining
Source: Microorganisms. 2020 Jan 30;8(2):190. doi: 10.3390/microorganisms8020190 (PMC7074749; doi:10.3390/microorganisms8020190)
Supplement: Supplementary file 1 [file microorganisms-08-00190-s001.pdf]

Keystone species and modularity in microbial hydrocarbon degradation uncovered by network analysis and association rule mining

Florian Centler [1,*], Sarah Günnigmann [1], Ingo Fetzer [2] and Annelie Wendeberg [1]

[1] UFZ – Helmholtz Centre for Environmental Research, Department of Environmental Microbiology, Permoserstraße 15, 04318 Leipzig, Germany; florian.centler@ufz.de

[2] Stockholm Resilience Centre, Stockholm University, Kräftriket 2B, 11419 Stockholm, Sweden; ingo.fetzer@stockholmresilience.su.se

* Correspondence: florian.centlerl@ufz.de; Tel.: +49-341-235-1336 (F.C.)

Supplementary Materials

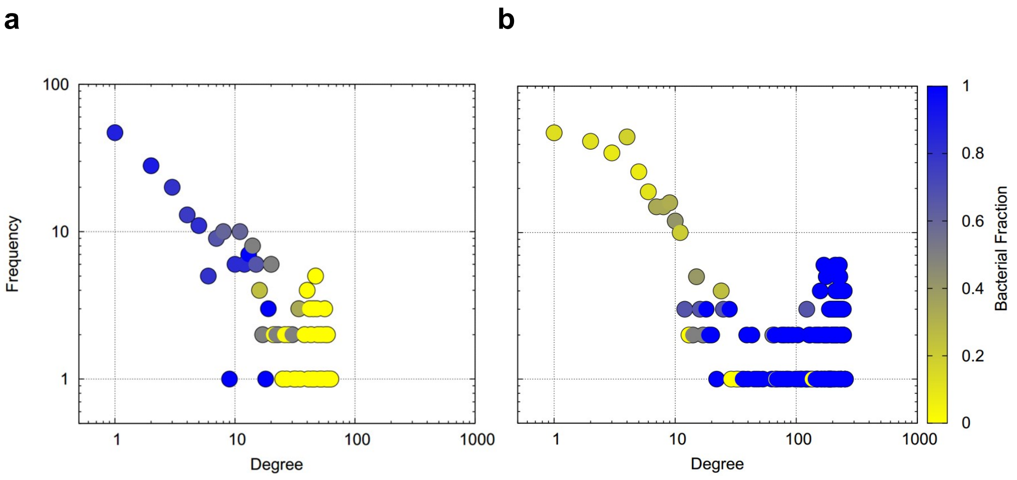

Figure S1. Degree distribution of co-occurrence networks. Color indicates fraction of bacterial fragments at respective degree levels, ranging from yellow indicating archaeal fragments only to blue indicating bacterial fragments only. Networks are based on *Hae*III digestion. (**a**) Network derived from bulk soil samples; (**b**) Network derived from single particle samples.

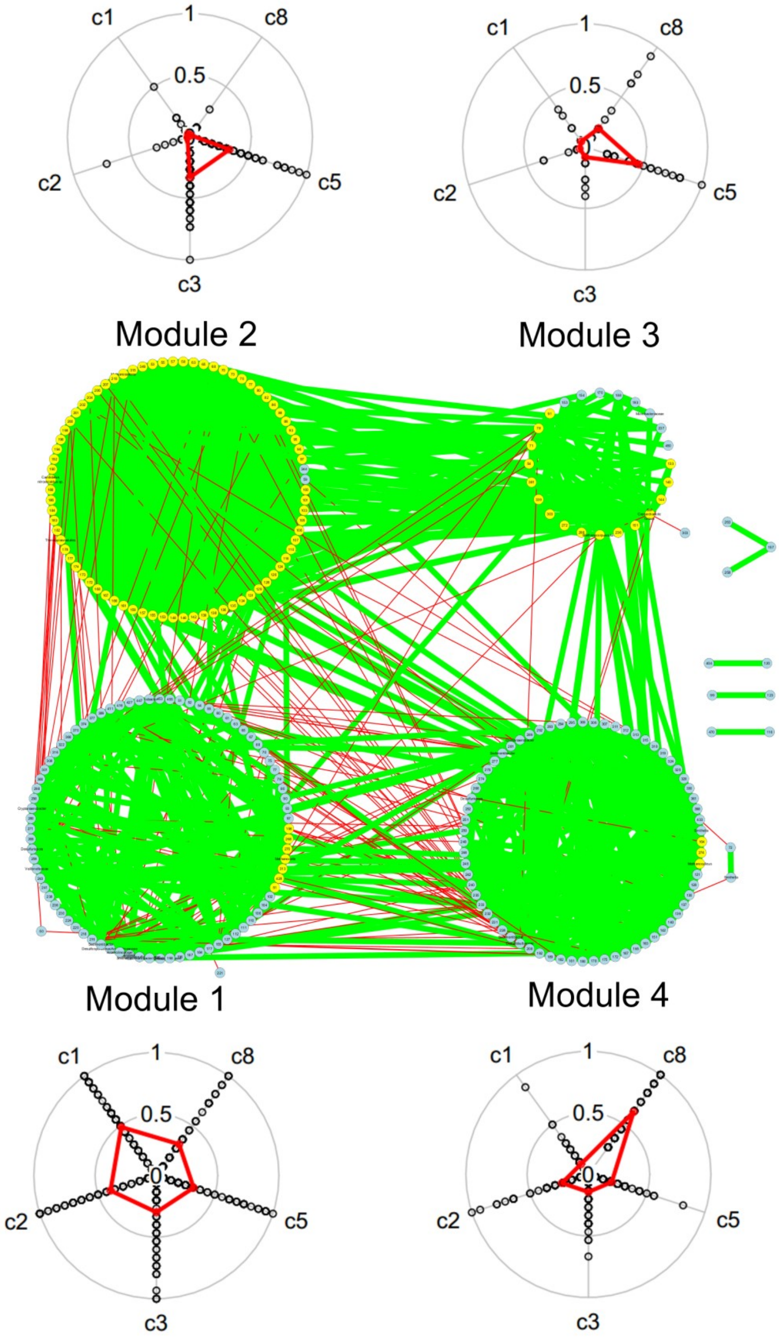

Figure S2. The co-occurrence network contains four modules whose presence along the flow path varies. The network is based on bulk soil samples and *Hae*III digestion. Bacterial fragments (light blue nodes) and archaeal fragments (yellow nodes) are connected by green edges for co-occurrence and thin red edges for mutual exclusion. Fragments belonging to modules as discovered by network analysis are arranged on circles. Modules' presence along the flow path (cores C1, C2, C3, and C5) and in pristine environment (C8) is shown in radial plots detailing the presence of individual module fragments (symbols) and module mean (red line), with values ranging from 0 (fragment not present in any core sample) and 1 (fragment present in all core samples). According to geochemical data, sulphate reduction was limited to core C1, while methanogenesis took place in cores C2, C3, and C5. See Table S2 for characterization of modules.

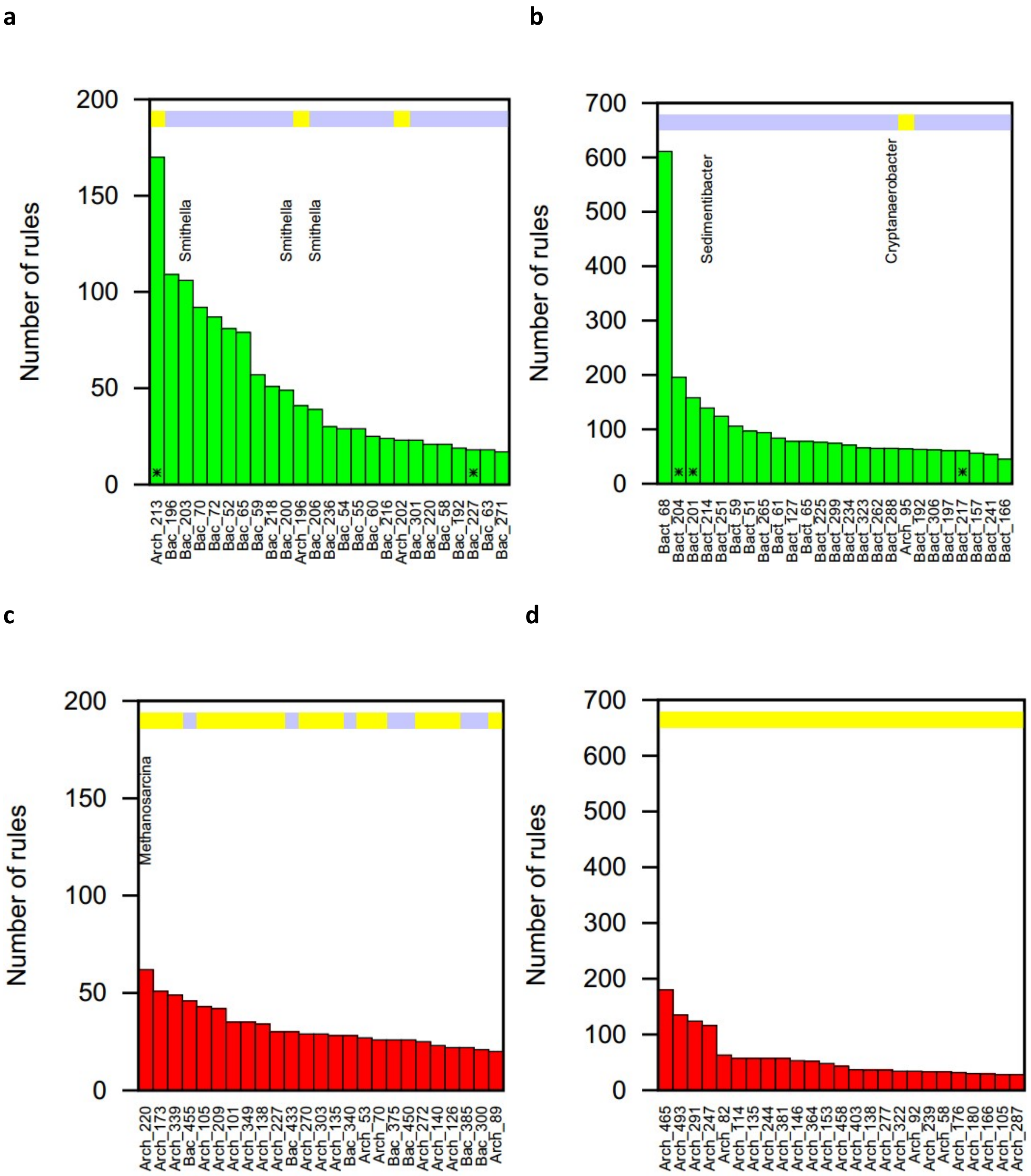

Figure S3. Hub nodes in association rule networks. Top 25 fragments exclusively participating in rules as targets or origins in the bulk and single particle data sets. Color bands indicate fragment type as bacterial (light blue) or archaeal (yellow). Networks are based on *Hae*III digestion. (**a**) Hub fragments acting as rule targets in the bulk data set; (**b**) Hub fragments acting as rule targets in the single particle data set[1]; (**c**) Hub fragments acting as rule origins in the bulk data set; (**d**) Hub fragments acting as rule origins in the single particle data set. Unambiguously identified fragments are noted, and bars marked with "*" had ambiguous identities: Arch_213: Methanoregula / Methanosaeta / Marine Group 1 Archaea; Bac_227: Gracilibacter / Bacillus / Microbacteriaceae; Bact_204: Smithella / Desulfobulbaceae; Bact_201: Smithella / Dehalobacter / Desulfobulbaceae; Bact_217: Proteiniphilum / Anaerolineaceae / Clostridiales.

[1] For the single particle data set only one fragment exclusively appeared as a rule target (participating in 611 rules). Hence, we relax this condition and show instead fragments for which the fraction of incoming links is above 0.95.

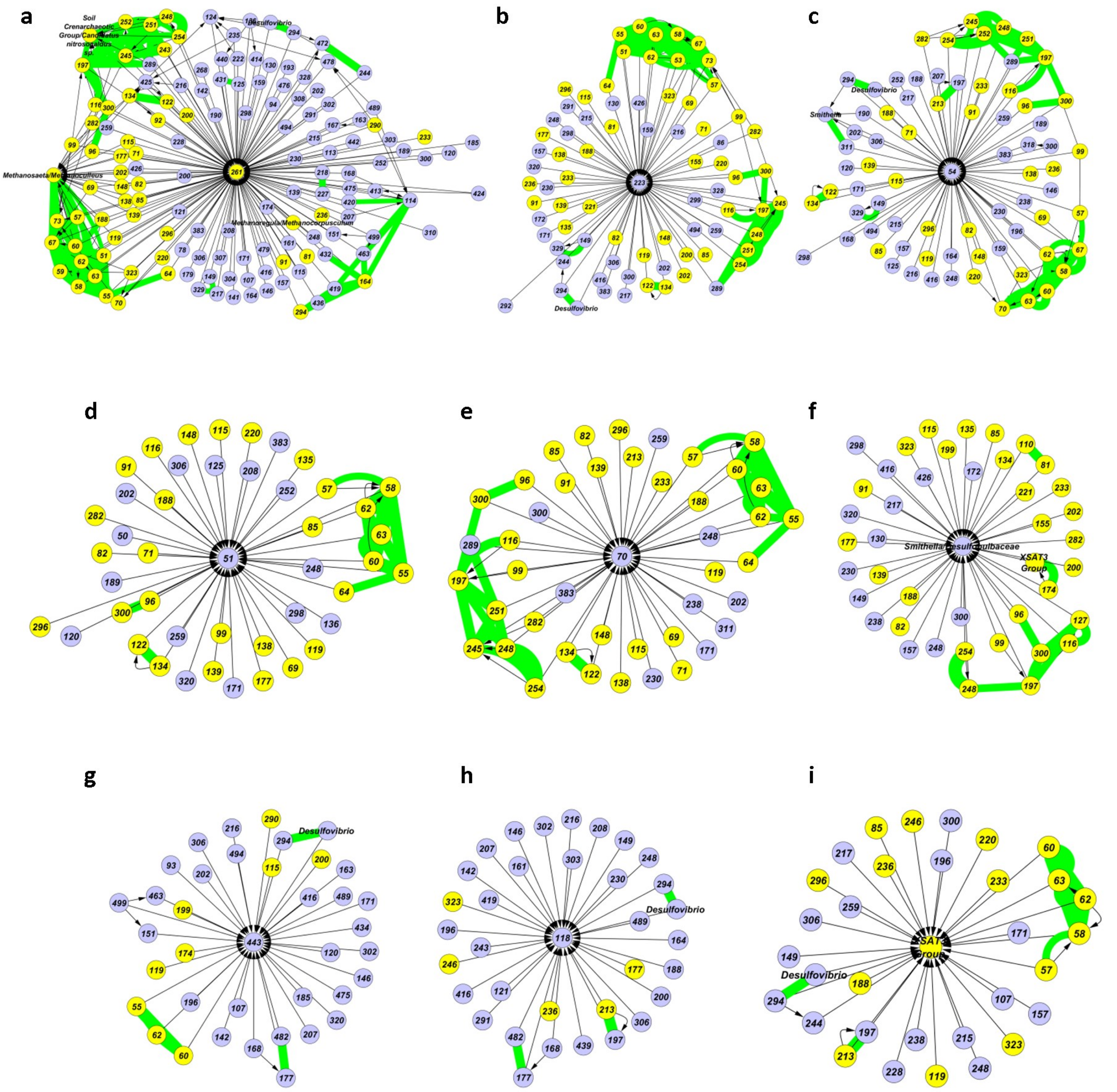

Figure S4. Hub nodes of the association rule network based on bulk data set. Showing all rule associations involving the central hub node fragment and directly neighboring fragments (arrows), and overlaying the co-occurrence network with green edges indicating co-occurrence relationships. Bacterial fragments are shown in light blue and archaeal fragments in yellow. Networks are based on *Rsa*I digestion. (**a-i**) The ten top hub nodes, with top 4th hub node shown in main text, Figure 4a.

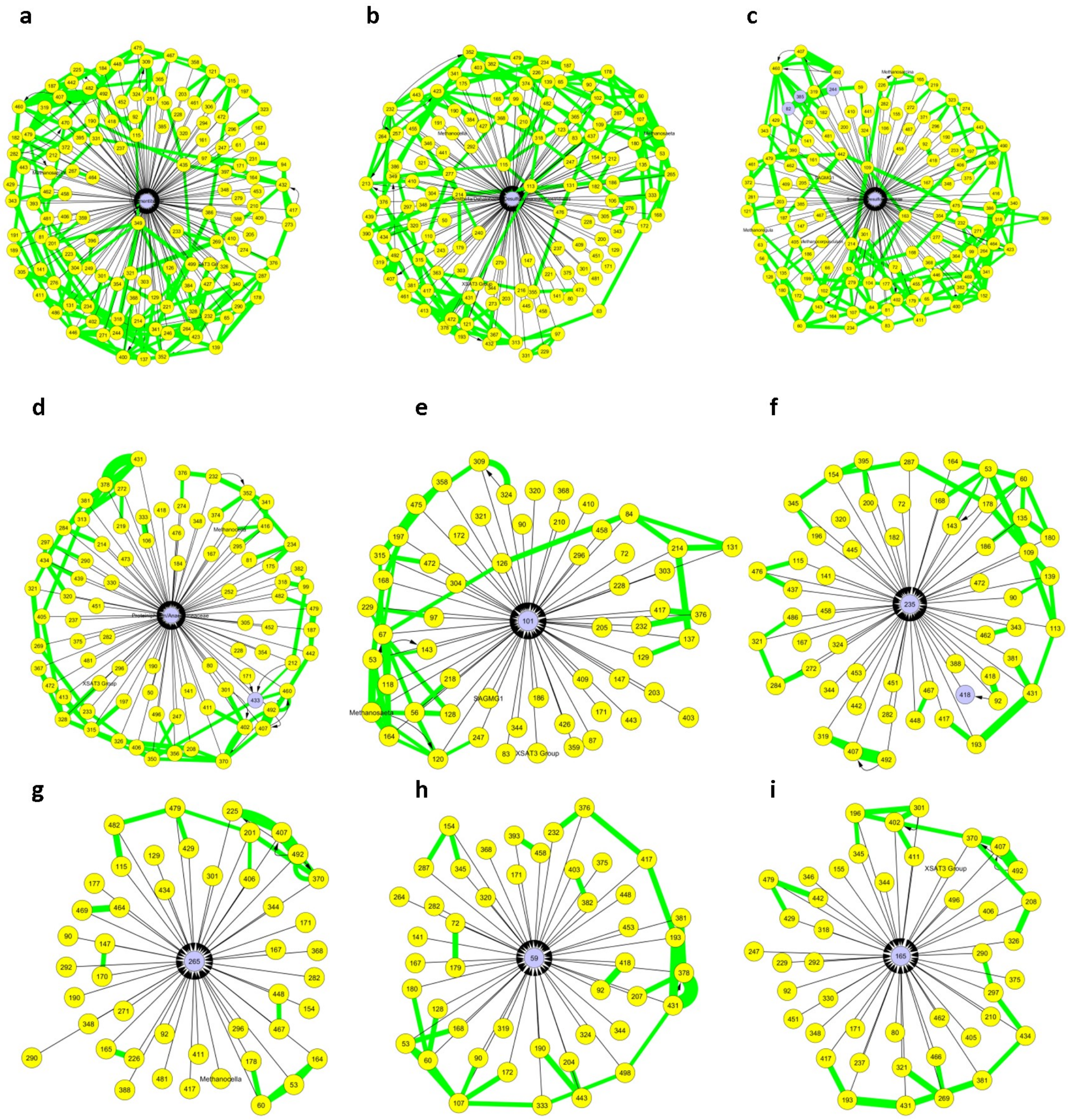

Figure S5. Hub nodes of the association rule network based on single particle data set. Showing all rule associations involving the central hub node fragment and directly neighboring fragments (arrows), and overlaying the co-occurrence network with green edges indicating co-occurrence relationships. Bacterial fragments are shown in light blue and archaeal fragments in yellow. Networks are based on *Rsa*I digestion. (**a-i**) The ten top hub nodes, with top 4th hub node shown in main text, Figure 4b.

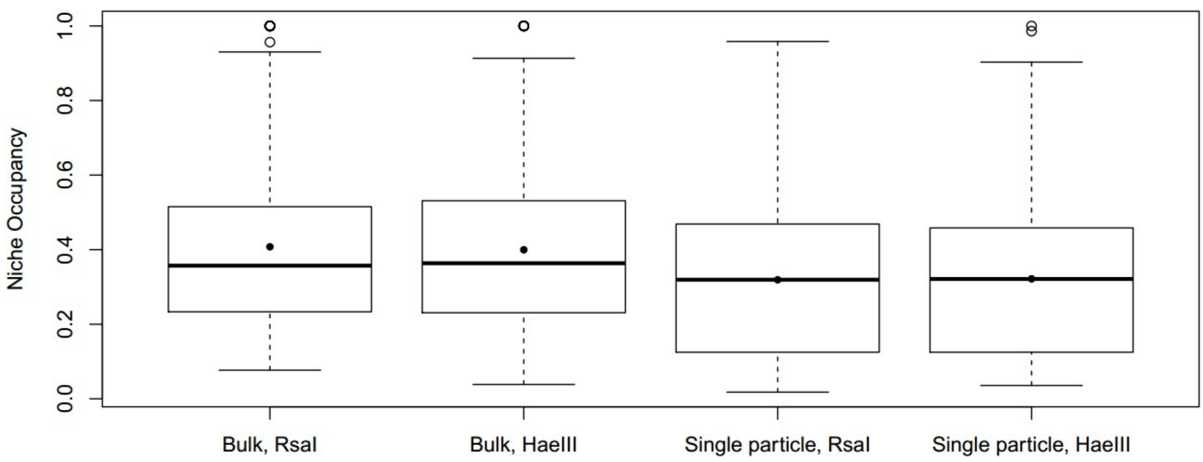

Figure S6. Distribution of fragments' niche occupancy in the four data sets. The extreme values regarding abiotic environmental concentrations (O2, MTBE, Benzene, and Toluene) in samples in which a fragment was detected were used to define its niche. Niche occupancy was then computed as the percentage of cases in which a fragment was present in samples which matched its niche as defined before. A value of 1 indicates that abiotic parameters alone can predict the presence of the fragment.

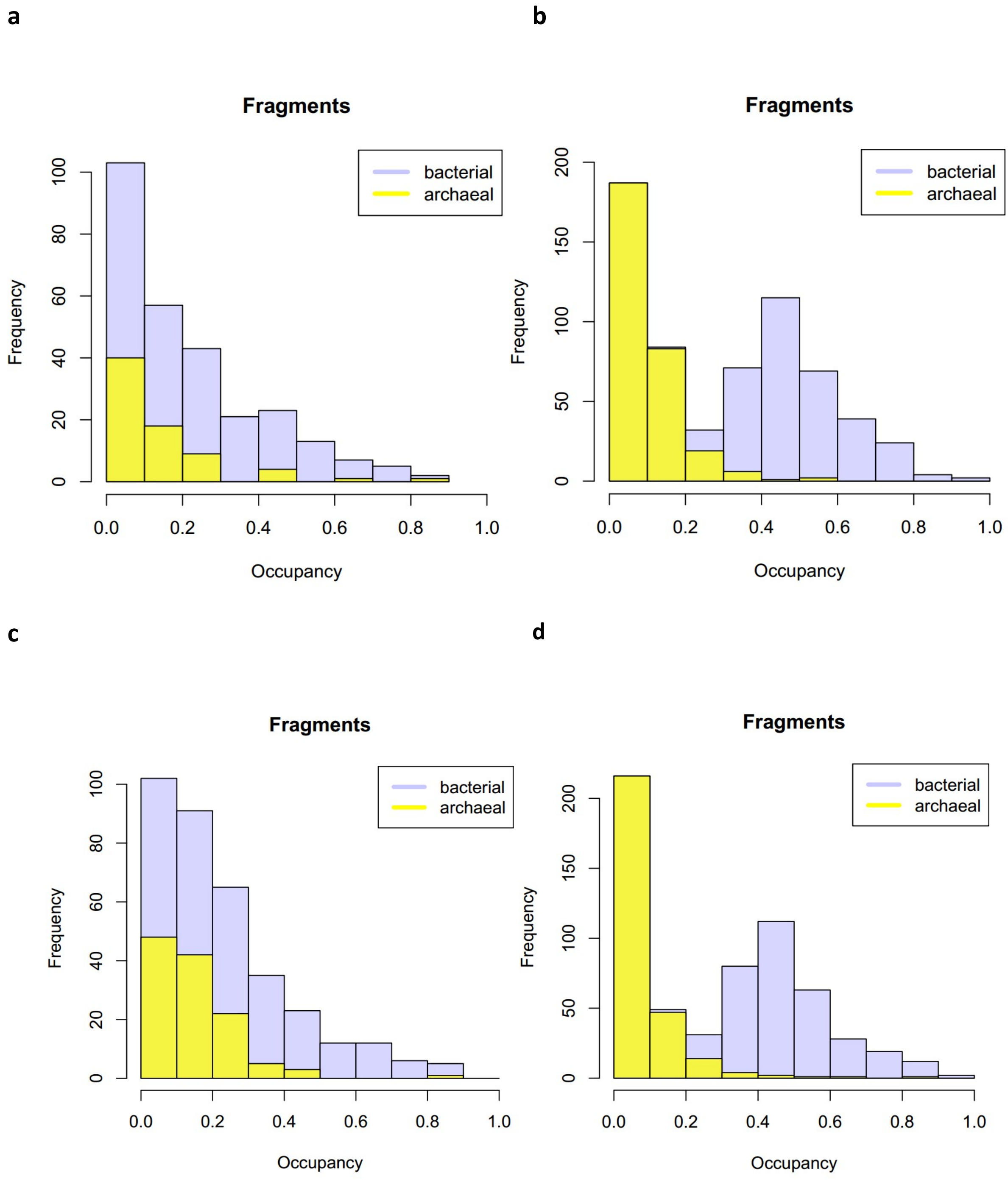

Figure S7. Distribution of fragment occupancy (presence in samples). (**a**) Bulk data set and *Rsa*I digestion; (**b**) Single particle data set and *Rsa*I digestion; (**c**) Bulk data set and *Hae*III digestion; (**d**) Single particle data set and *Hae*III digestion. Note that in the single particle data sets, the frequencies for bacterial and archaeal fragments are identical for the lowest occupancy level.

Table S1. Characterization of modules containing more than 3 fragments, *Rsa*I digestion.

	Composition		Interactions				Occupancy[1]	
Id	Fragments	Bacterial Fraction	Total	Positive	Negative	Density	Samples	Standard Deviation
1	16	0.75	34	34	0	0.28	21.2	11.9
2	20	0.8	23	23	0	0.12	32.9	15.5
3	17	0.06	72	72	0	0.53	14.1	8.2
4	13	0.08	30	30	0	0.38	7.9	3.4
5	21	0.90	35	35	0	0.17	16.8	7.7

[1] number of samples in which module was present, calculated as the average fragment occupancy (number of samples in which a fragment was present) of all fragments belonging to module

Table S2. Characterization of modules containing more than 3 fragments, *Hae*III digestion.

	Composition		Interactions				Occupancy[2]	
Id	Fragments	Bacterial Fraction	Total	Positive	Negative	Density[1]	Samples	Standard Deviation
1	80	0.91	173	162	11	0.05	26.3	15.2
2	78	0.03	1,223	1,223	0	0.41	11.3	7.0
3	24	0.33	42	42	0	0.15	12.2	8.6
4	68	0.96	218	218	0	0.10	17.1	8.8

[1] only considering positive interactions; [2] number of samples in which module was present, calculated as the average fragment occupancy (number of samples in which a fragment was present) of all fragments belonging to module
